# *CABLES1* expression is reduced in human subcutaneous adipose tissue in obesity and type 2 diabetes but may not directly impact adipocyte glucose and lipid metabolism

**DOI:** 10.1080/21623945.2023.2242997

**Published:** 2023-08-09

**Authors:** Susanne Hetty, Milica Vranic, Prasad G Kamble, Martin H Lundqvist, Maria J Pereira, Jan W Eriksson

**Affiliations:** a Department of Medical Sciences, Clinical Diabetes and Metabolism, Uppsala University, Uppsala, Sweden; b Innovation Strategies & External Liaison, Pharmaceutical Technologies & Development, AstraZeneca R&D, Mölndal, Sweden

**Keywords:** CABLES1, type 2 diabetes, obesity, adipose tissue, adipocytes

## Abstract

Cdk5 and Abl enzyme substrate 1 (CABLES1) is a cell cycle regulator that has previously been identified as a candidate gene for obesity-related phenotypes, but little is known about its role in adipose tissue metabolism. In this study, we explore the role of CABLES1 in obesity and type 2 diabetes (T2D) in human subcutaneous adipose tissue (SAT). We performed gene expression analysis of SAT obtained from subjects with and without T2D, and from a second validation cohort consisting of subjects without T2D. We used CRISPR/Cas9 genome editing to perform CABLES1 loss-of-function studies in human primary preadipocytes and assessed them functionally after differentiation. *CABLES1* gene expression in SAT was decreased in T2D by almost 25%, and inversely associated with insulin resistance markers and hyperglycaemia. mRNA levels were reduced with increasing BMI and negatively correlated with obesity markers. We found that adipocytes are likely the main CABLES1-expressing cell type in SAT, but CABLES1 depletion in adipocytes caused no phenotypical changes in regards to differentiation, glucose uptake, or expression of key genes of adipocyte function. These findings suggest that *CABLES1* gene expression in SAT might be altered in obesity and T2D as a consequence of metabolic dysregulation rather than being a causal factor.

## Introduction

Obesity, an accumulation of excess adipose tissue (AT), is often accompanied by insulin resistance, which contributes to the development of serious metabolic disorders, including type 2 diabetes (T2D) and cardiovascular disease [1]. Besides providing a storage site for fat, AT is a key organ in the metabolic regulation of insulin-target tissues, such as skeletal muscle and liver [2]. AT exerts this effect principally through the release of bioactive molecules, such as free fatty acids, adipokines and cytokines [3]. In the obese state, AT is often dysregulated regarding glucose and lipid metabolism, as well as the production and release of these molecules. Besides the accumulation of an excess of total body fat, the distribution of fat plays an essential role in the development of cardiometabolic diseases, where accumulation in abdominal depots leads to a worse metabolic outcome compared to fat stored peripherally [1,4]. Therefore, identifying factors that impact AT function and distribution is important to understand underlying molecular mechanisms and to find potential targets for the treatment and prevention of obesity and T2D.

Cdk5 and Abl enzyme substrate 1 (CABLES1; encoded by the *CABLES1* gene), also called IK3–1, is a cyclin-dependent kinase (CDK)-binding protein that regulates the activity of several CDKs, such as Cyclin-dependent kinase 2 (CDK2) and Cyclin-dependent kinase 5 (CDK5), by interacting with non-receptor tyrosine kinases, and can thereby affect cell differentiation and proliferation [5,6]. The loss of CABLES1 leads to increased proliferation in many cell types and has been linked to several cancers [7–9]. Cell cycle regulation is important for preadipocyte proliferation and adipogenesis, and factors involved in these cellular processes have been shown to impact adipocyte function [10–12]. CABLES1 also acts as an adaptor protein that enhances CDK5 tyrosine phosphorylation which activates CDK5 by promoting interaction with its activating subunit p35 [5]. Activated CDK5 can in turn phosphorylate proliferator of peroxisome-activated receptor gamma (PPARG), a master regulator of adipogenesis and adipocyte metabolism, at serine 273 in AT, as seen in diet-induced obesity in mice [13]. This phosphorylation of PPARG dysregulates the expression of numerous PPARG-regulated genes, including the gene *ADIPOQ* coding for the key *in vivo* insulin-sensitizing adipokine adiponectin, and has therefore been linked to the development of insulin resistance [14]. Moreover, *CABLES1* was identified in a genome-wide association study (GWAS) as a gene associated with obesity-related phenotypical traits [15,16], and was recently proposed as a top candidate, adipose tissue-specific, obesity-risk regulatory SNP-containing gene in an epigenomic and transcriptomic GWAS meta-analysis study [17]. However, whether CABLES1 *per se* plays a role in adipose tissue metabolism is unknown.

The aim of this study was to investigate the role of *CABLES1* in AT metabolism in T2D and obesity, and its potential functional role in adipocyte development and metabolism. We used RNAseq analysis of subcutaneous adipose tissue (SAT) from subjects without and with T2D, group-matched for age and BMI, to study the associations of *CABLES1* gene expression with clinical markers of obesity and insulin resistance, as well as genes involved in adipose tissue function. We used a second cohort of subjects without T2D to validate the associations. Moreover, we performed CRISPR/Cas9 knockdown of the *CABLES1* gene in primary human preadipocytes to functionally characterize its role in adipogenesis and lipid and glucose metabolism.

## Results

### CABLES1 gene expression was reduced in SAT from subjects with T2D, and was associated with markers of hyperglycaemia and insulin resistance

**Figure 1.**
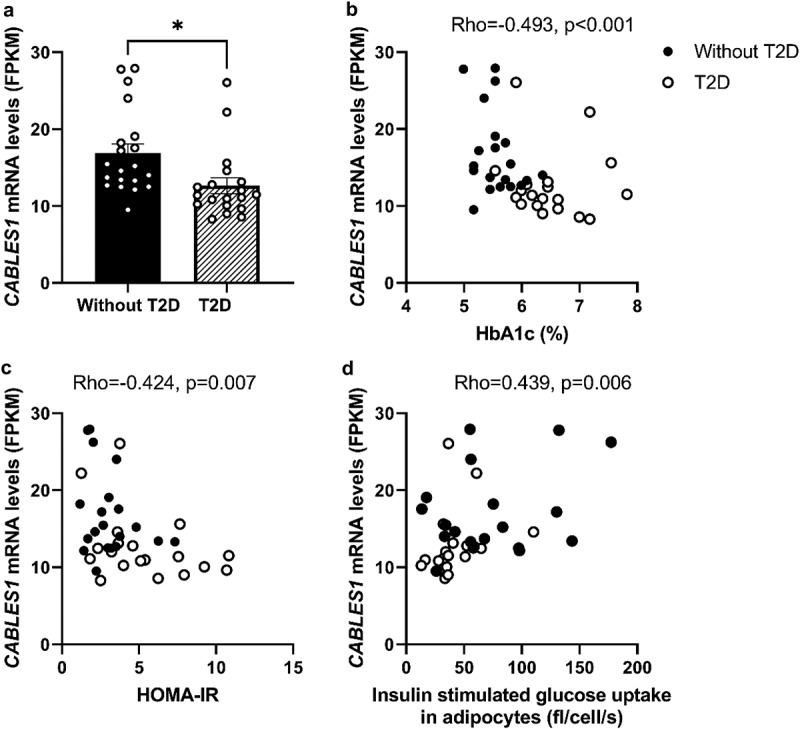
***CABLES1* gene expression is decreased in SAT from T2D compared to subjects without T2D, and correlated with markers of hyperglycaemia and insulin resistance**. *CABLES1* gene expression in a) SAT from BMI- and age-matched subjects without (*n* = 20) and with T2D (*n* = 19) using RNAseq. Correlations between *CABLES1* gene expression in SAT and b) HbA1c c) HOMA-IR, and d) insulin (1000 μU/mL) stimulated glucose uptake in isolated adipocytes *ex vivo* (*cohort 1*). Data represents mean ± SEM. *p < 0.05.

Note: HOMA-IR, homoeostatic model assessment of insulin resistance index; SAT, Subcutanous adipose tissue; T2D, type 2 diabetes; HbA1c, glycosylated haemoglobin.

Clinical characteristics for all subjects are shown in Table 1. In the RNAseq gene expression analysis (*cohort 1*), *CABLES1* mRNA levels were found to be significantly decreased by approximately 25% (*p* < 0.05) in SAT from patients with T2D (*n* = 19) compared to subjects without T2D (*n* = 20) (Figure 1(a)). Associations between *CABLES1* mRNA levels in SAT with clinical and metabolic variables were explorative and are shown in Table 2. In *cohort 1*, gene expression of *CABLES1* in SAT was negatively associated with HbA1c, HOMA-IR, fasting glucose, glucose area under the curve (AUC) during OGTT, insulin and C-peptide, and positively with Matsuda index and *ex vivo* basal and insulin-stimulated glucose uptake (Table 2, Figure 1(b–d)). Similar correlations for hyperglycaemia and insulin resistance markers were seen for *cohort 2*, with the exception of fasting glucose and HbA1c, which could be explained by the narrower range for these parameters in this cohort, consisting of subjects without T2D.

**Table 1.** Anthropometric and clinical characteristics of study participants.

	*Cohort 1*^a^	*Cohort 2*	*Cohort 3*^b^
N	39	102	12
T2D	19		12
Men/Women (number)	19/20	27/75	3/9
Age (years)	57 ± 10	49 ± 18	50 ± 9
Plasma glucose (mmol/L)	6.0 ± 0.7	5,7 ± 1.0	8.5 ± 1.8
Serum insulin (mU/L)	13.7 ± 6.4	10.4 ± 7.5	28.8 ± 11.3
Serum C-Peptide (nmol/L)	1.00 ± 0.34	0.82 ± 0.43	1.60 ± 0.31
HbA1C, IFCC (mmol/mol)	42.3 ± 7.4	34.9 ± 4.1	56.1 ± 11.9
HOMA-IR	4.2 ± 2.6	2.8 ± 2.3	10.5 ± 4.0
Matsuda	3.35 ± 1.92	NA	1.31 ± 0.43
Plasma total cholesterol	5.3 ± 1.2	5.1 ± 1.0	4.6 ± 0.9
Plasma HDL-cholesterol (mmol/L)	1.2 ± 0.3	1.5 ± 0.4	1.0 ± 0.2
Plasma LDL-cholesterol (mmol/L)	3.4 ± 1.0	3.1 ± 0.9	2.9 ± 0.8
Trigycerides (mmol/L)	1.6 ± 0.6	1.6 ± 0.6	2.2 ± 1.0
OGTT AUC Glucose (min*mmol/L)	1918 ± 669	NA	2514 ± 637
OGTT AUC insulin (min*mU/L)	9520 ± 5325	NA	15507 ± 7605
OGTT AUC FFA (min*µmol/L)	26995 ± 7207	NA	26795 ± 9212
BMI (kg/m [2]	30.7 ± 4.7	29.0 ± 9.0	36.2 ± 3.3
Waist-hip-ratio	0.97 ± 0.06	0.90 ± 0.10	0.98 ± 0.06
SAT volume (mL/kg_bw_)	50.5 ± 17.0	NA	NA
VAT volume (mL/kg_bw_)	36.2 ± 10.7	NA	NA
Liver fat (%)	11.1 ± 8.4	NA	NA

Note: Data presented as mean ± SD. Blood chemistry is fasting.

BMI, body mass index; HbA1c, glycosylated haemoglobin; FFA, free-fatty acids; HOMA-IR, homoeostatic model assessment of insulin resistance index; HDL, high-density lipoprotein; LDL, low-density lipoprotein; NA, not available; OGTT AUC, oral-glucose-tolerance test, area under the curve; SAT, subcutaneous adipose tissue; VAT, visceral adipose tissue.

^a^Full cohort details published previously [25].

^b^Only pre-RYGB surgery (baseline) values shown. Full cohort details, and study design, with clinical characteristics at all time points published previously [26,27].

**Table 2.** Correlations between *CABLES1* and clinical and metabolic parameters.

	Cohort 1		Cohort 2	
	*n* = 39		*n* = 67–90	
	*Rho*	*p-value*	*Rho*	*p-value*
*Clinical characteristics*				
Age	0.052	0.754	0.027	0.797
Weight	−0.050	0.761	**−0.487**	<0.001
BMI	−0.040	0.810	**−0.471**	<0.001
Waist	−0.103	0.532	**−0.456**	<0.001
Hip	0.042	0.801	**−0.422**	<0.001
Waist-hip-ratio	−0.210	0.200	**−0.244**	0.021
SAT volume	0.060	0.736	NA	
VAT volume	−0.286	0.101	NA	
SAT/VAT volume	−0.152	0.391	NA	
Liver fat %	−0.187	0.291	NA	
*Hyperglycemia and insulin resistance markers*				
HbA1C	**−0.493**	0.001	−0.120	0.261
HOMA-IR	**−0.424**	0.007	**−0.404**	<0.001
Fasting glucose	**−0.551**	<0.001	−0.147	0.166
Fasting insulin	**−0.344**	0.032	**−0.457**	<0.001
Fasting C-peptide	**−0.330**	0.040	**−0.447**	<0.001
HDL-cholesterol	*0.294*	0.069	**0.465**	<0.001
LDL-cholesterol	0.134	0.416	−0.087	0.417
HDL/LDL ratio	−0.083	0.615	**−0.397**	<0.001
Triglycerides	*-0.292*	0.071	**−0.301**	0.004
OGTT AUC glucose	**−0.570**	<0.001	NA	
OGTT AUC insulin	0.057	0.729	NA	
OGTT AUC FFA	**−0.376**	0.018	NA	
OGTT AUC Glycerol	−0.011	0.943	NA	
Matuda	**0.392**	0.014	NA	
*SAT adipocytes ex vivo*				
Lipolysis basal	−0.173	0.291	−0.171	0.457
Lipolysis maximal stimulation^a^	**0.403**	0.011	−0.099	0.670
Glucose uptake basal	**0.357**	0.028	**0.351**	0.015
Glucose uptake insulin^b^	**0.439**	0.006	**0.372**	0.009
Subcutaneous adipocyte size	−0.108	0.514	**−0.282**	0.015

Note: Table shows Spearman’s rho-correlation coefficients. Significances shown as bold *p* < 0.05, italics *p* < 0.1.

BMI, body mass index; HbA1c, glycated haemoglobin; HDL, high-density lipoprotein; HOMA- IR, Homoeostatic model assessment; LDL, low-density lipoprotein; NA, not available; OGTT AUC, oral-glucose-tolerance test, area under the curve; SAT, subcutaneous adipose tissue; VAT, visceral adipose tissue WHR, Waist-to-hip ratio.

^a^Maximal lipolysis with 0.5 μM isoprenaline.

^b^Glucose uptake with 1000 µU/mL of insulin.

### CABLES1 gene expression in SAT was reduced in obesity and was correlated with markers of obesity

**Figure 2.**
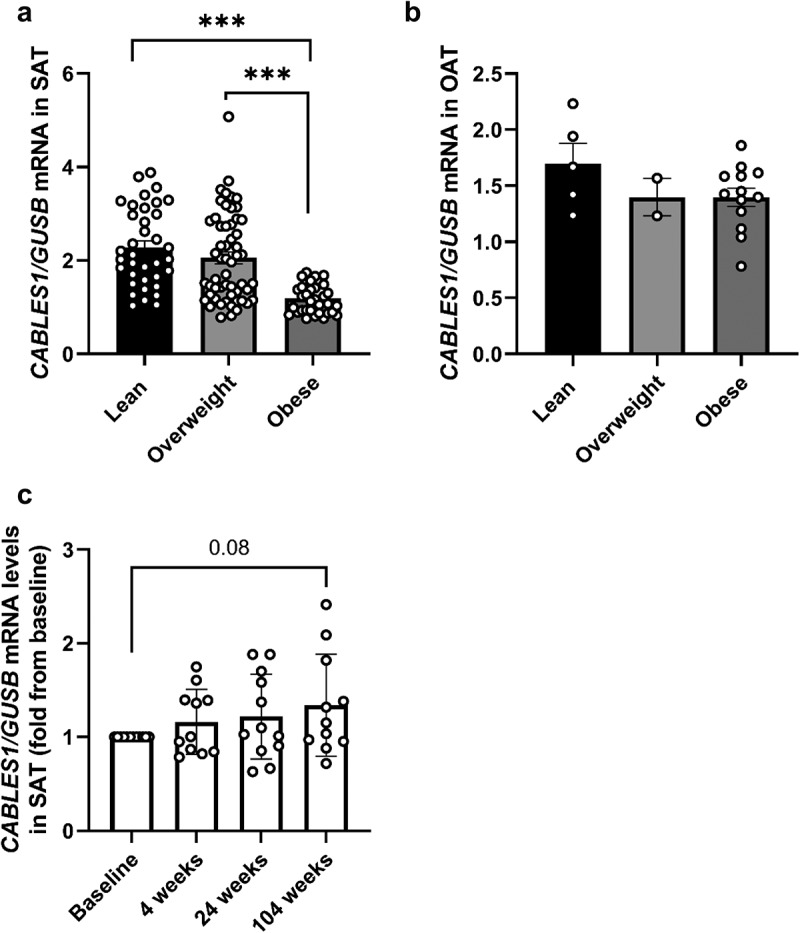
***CABLES1* gene expression in SAT is decreased in obesity**. *CABLES1* gene expression in a) SAT from lean (*n* = 36), overweight (*n* = 55) and obese (*n* = 34) subjects, and in b) OAT from lean (*n* = 5), overweight (*n* = 2) and obese (*n* = 13) subjects (A-B: *cohort 2–3*), and in c) subjects in SAT before (baseline), 4, 24 and 104 weeks after RYGB surgery (*n* = 12) (*cohort 3*). Data represents mean ± SEM.*** p*<*0.001.

Note: OAT, Omental adipose tissue; SAT, Subcutaneous adipose tissue.

To assess the effect of adiposity, we compared the expression of *CABLES1* in SAT and OAT from non-diabetic lean (BMI <25 kg/m^2^, *n* = 36/5, SAT/OAT), overweight (BMI 25–30 kg/m^2^, *n* = 55/2, SAT/OAT) and obese individuals (BMI >30 kg/m^2^, *n* = 34/13, SAT/OAT) (Figure 2(a,b)). In SAT, *CABLES1* gene expression was 50% (*p* < 0.001) lower in subjects with obesity compared to lean (Figure 2(a)). Additionally, in subjects with obesity, *CABLES1* gene expression was reduced by approximately 40% (*p* < 0.001) compared to subjects with overweight. In OAT, *CABLES1* gene expression was lower in overweight and obese compared to lean, but this did not reach significance (Figure 2(b)). Moreover, in *cohort 3*, which consisted of subjects with obesity that underwent RYGP surgery, there was a trend for increased *CABLES1* gene expression by 34% at 104 weeks following RYGP surgery compared to baseline levels (*p* = 0.08) (Figure 2(c)). At baseline in *cohort 3*, *CABLES1* was negatively correlated with weight (rho −0.594, *p* = 0.042), and there was a trend for a negative correlation at 24 weeks post-surgery between the delta change in *CABLES1* gene expression and the amount of weight lost, indicating that the greater the weight loss the more increase in *CABLES1* expression (rho −0.511, *p* = 0.089). No difference in *CABLES1* gene expression was found between females vs. males in SAT (Supplementary Figure S1).

For *cohort 1*, we did not see any correlations between adiposity markers and *CABLES1* mRNA levels besides a negative correlation with free fatty acids (FFA) AUC during OGTT (Table 2). The correlations were repeated in *cohort 2*, covering a broader range of BMI (20–58 kg/m^2^, *n* = 91), where *CABLES1* gene expression correlated inversely with body weight, BMI, WHR, plasma triglycerides, LDL/HDL ratio, and adipocyte size (Table 2). In addition, a positive correlation was found between *CABLES1* gene expression and plasma HDL. Age did not correlate with *CABLES1* gene expression in either cohort (Table 2). When key significantly associated variables in the correlation analysis for *cohort 2* were entered into a multivariate regression analysis model, only BMI (Standardized *β* coefficient −0.328, *p* < 0.029) remained significantly associated with *CABLES1* mRNA levels in SAT (Table 3). Sex was included in the regression analysis but was not a significant predictor of *CABLES1* levels. In order to exclude a batch effect due to biopsy methodology (needle vs. surgery) and hospital where the biopsies were obtained, we performed regression analysis including BMI and either of these parameters as predictors, but only BMI came out significant (both models *p* < 0.001 for BMI). Both biopsy methods showed similar significant correlations between *CABLES1* gene expression and BMI (Supplementary Fig S2).

**Table 3.** Multilinear regression analysis of *CABLES1* mRNA levels in SAT and clinical markers of obesity and insulin resistance in subjects without T2D (*cohort 2*.).

	R^2^	*Standardized β*	*t*	95% CI	p-value
**Model**	0.246				<0.001
HOMA-IR		−0.201	−1.341	(−0.191, 0.037)	0.184
BMI		**−0.328**	**−2.217**	**(−0.058, −0.003)**	0.029
WHR		−0.023	−0.205	(−2.251, 1.831)	0.838
Sex		0.125	1.182	(−0.171, 0.070)	0.241

Note: Significances shown as bold, *p* < 0.05. *N* = 90.

BMI, body mass index; CI, confidence interval CI, confidence interval for unstandardized coefficient (lower bound, upper bound); HOMA- IR, Homoeostatic model assessment; WHR, Waist-hip ratio.

### CABLES1 is mainly expressed in adipocytes in SAT

**Figure 3.**
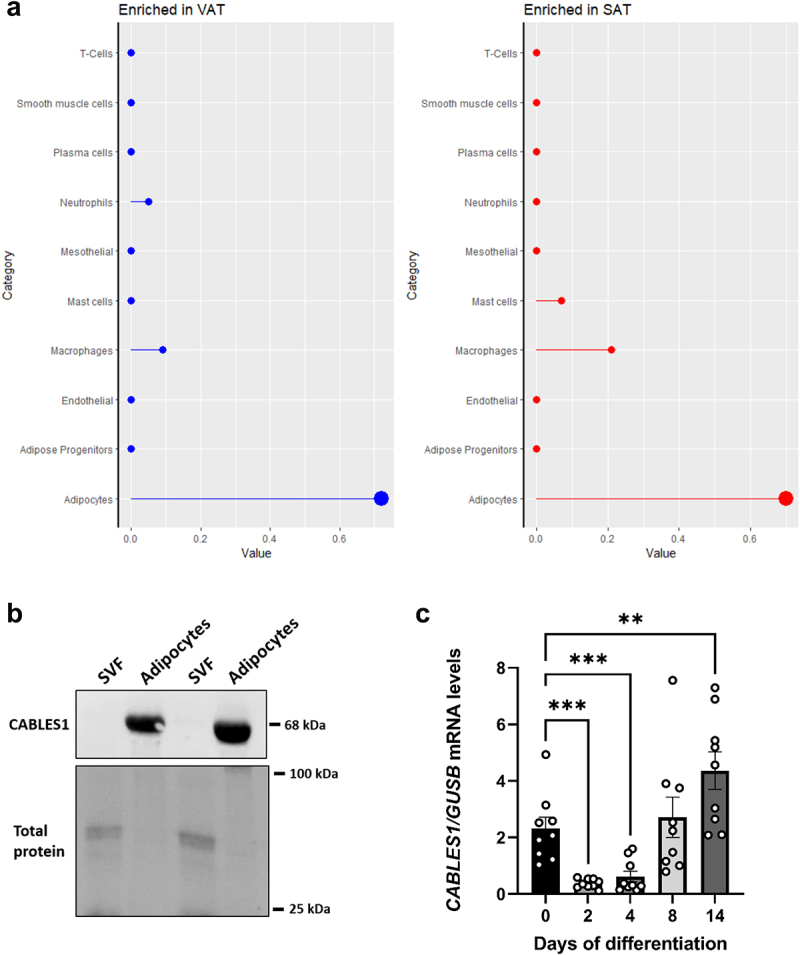
**CABLES1 expression in SAT and VAT, and during preadipocyte differentiation**. a) CABLES1 enrichment prediction score across cell types. This score is the mean correlation between the gene and the three reference transcripts selected to represent each cell type profiled within Visceral (VAT, left) and Subcutaneous adipose (SAT, right). A dot indicates a positive correlation and the larger circle symbol classifies CABLES1 as cell type enriched in adipocytes. A mean correlation value with adipocytes is defined by correlation with the expression of the three adipocyte reference transcripts: *ADIPOQ*, *LIPE*, *PLIN1*. Simultaneous positive correlation with all three transcripts is cell type enrichment. Non positive correlations are shown as 0. Data from doi: 10.1016/j.Celrep.2022.11104. b) Representative immunoblot of CABLES1 protein and total protein levels in adipocytes and SVF (*n* = 2). c). *CABLES1* gene expression levels on day 0 (preadipocytes), 2, 4, 8 and 14 (mature adipocytes) of differentiation, measured by qPCR (*n* = 8). The qPCR gene expression data was normalized using *GUSB* as a reference gene. All data are shown as mean ± SEM. **p < 0.01, ***p < 0.001.

In order to investigate the relative contribution from different cell types in SAT and VAT to levels of *CABLES1* in this tissue, we performed gene enrichment analysis of single cell transcriptomics data using publicly available databases, which showed that *CABLES1* is enriched in adipocytes in both SAT and VAT, but that *CABLES1* can also be found in other cells types such as macrophages (SAT) and mast cells (VAT), (Figure 3(a)). Also, western blot analysis of CABLES1 protein in SVF and adipocytes isolated from SAT showed that CABLES1 was robustly expressed in adipocytes but below quantification level in SVF (Figure 3(b)). Moreover, we measured gene expression levels in SVF-derived preadipocytes before initiation of differentiation (day 0), and after 2, 4, 8 and 14 days of differentiation. Preadipocytes expressed *CABLES1 in vitro*, and, while levels were transiently reduced on day 2 and 4, they were significantly higher (2-fold compared to day 0) after 14 days of differentiation, when the cells have become mature adipocytes (Figure 3(c)).

### CABLES1 gene expression was associated with genes involved in adipocyte differentiation, lipid and glucose metabolism, and cell cycle regulation

*CABLES1* gene expression in SAT was associated with measures of insulin sensitivity, adiposity and lipids, and therefore we further investigated whether *CABLES1* correlated with genes involved in adipocyte development and metabolism (*cohort 1*) (Table 4). *CABLES1* gene expression in SAT was positively associated with key genes involved in adipocyte differentiation and lipogenesis, such as *PPARG, FASN* (both *p* < 0.05), *CEBPA* (*p* < 0.01), *CIDEC* (*p* < 0.001), whereas it was negatively associated with *CD36* (*p* < 0.05). Moreover, *CABLES1* gene expression was positively correlated with genes important for lipolysis, such as *ATGL, MGLL, PLIN4* (all *p* < 0.001), *LIPE* (*p* < 0.01) and *ADRA2A* (*p* < 0.05), and genes important for cell cycle regulation; *CDK5, CDKN2C* (both *p* < 0.05) and *CDKN1A* (*p* < 0.001). No correlations were found between mRNA levels of *CABLES1* and the adipokines *LEP* or *ADIPOQ* (Table 4).

**Table 4.** Correlations between *CABLES1* gene expression in SAT and genes involved in adipogenesis and adipocyte function.

	Cohort 1 (*n* = 39)	
	*Rho*	*p-value*
*Adipogenesis*		
*PPARG*, Peroxisome proliferator-activated receptor gamma	**0.395**	0.013
*CEBPA*, CCAAT enhancer-binding protein alpha	**0.443**	0.005
*FABP4*, Fatty acid binding protein 4	*0.304*	0.060
*LEP*, Leptin	0.250	0.124
*ADIPOQ*, Adiponectin	−0.053	0.749
*Lipogenesis*		
*AGPAT1*, 1-Acylglycerol-3-Phosphate O-Acyltransferase 1	**0.583**	<0.001
*AGPAT2*, 1-Acylglycerol-3-Phosphate O-Acyltransferase 2	**0.589**	<0.001
*AGPAT3*, 1-Acylglycerol-3-Phosphate O-Acyltransferase 3	**0.409**	0.010
*DGAT1*, diacylglycerol acyltransferase-1	**0.520**	<0.001
*DGAT2*, diacylglycerol acyltransferase-2	*0.304*	0.060
*CIDEC*, Cell death inducing DFFA like effector C	**0.511**	<0.001
*FASN*, Fatty acid synthas	**0.332**	0.039
*LPL*, lipoprotein lipase	−0.250	0.880
*CD36*, Cluster of differentiation 36	**−0.431**	0.039
*Lipolysis*		
*ATGL*, Adipose triglyceride lipase	**0.570**	<0.001
*HSL*, Hormone-sensitive lipase	**0.456**	0.003
*MGLL*, Monoglyceride lipase	**0.604**	<0.001
*ADRA2A*, Adrenoceptor alpha 2A	**0.334**	0.038
*PLIN3*, Perilipin 3	**0.402**	0.011
*PLIN4*, Perilipin 3	**0.518**	<0.001
*Glucose uptake*		
*SLC2A1*, Glucose transporter type 1	0.253	0.120
*SLC2A4*, Glucose transporter type 4	**0.439**	0.005
*AKT1*, AKT Serine/Threonine Kinase 1	**0.494**	0.001
*AKT2*, AKT Serine/Threonine Kinase 1	0.230	0.160
*INSR*, insulin receptor	**0.334**	0.038
*Cell cycle*		
*CDK2*, Cyclin Dependent Kinase 2	−0.164	0.317
*CDK5*, Cyclin Dependent Kinase 5	**0.395**	0.013
*CDKN1A*, Cyclin Dependent Kinase Inhibitor 1A	**0.550**	<0.001
*CDKN1B*, Cyclin Dependent Kinase Inhibitor 1B	*-0.313*	0.053
*CDKN2C*, Cyclin Dependent Kinase Inhibitor 2C	**0.351**	0.028

Note: Table shows Spearman’s rho-correlation coefficients. Significances shown as bold, *p* < 0.05 and italics, *p* < 0.1.

### Ablation of CABLES1 in human primary preadipocytes did not affect adipocyte differentiation or glucose uptake

**Figure 4.**
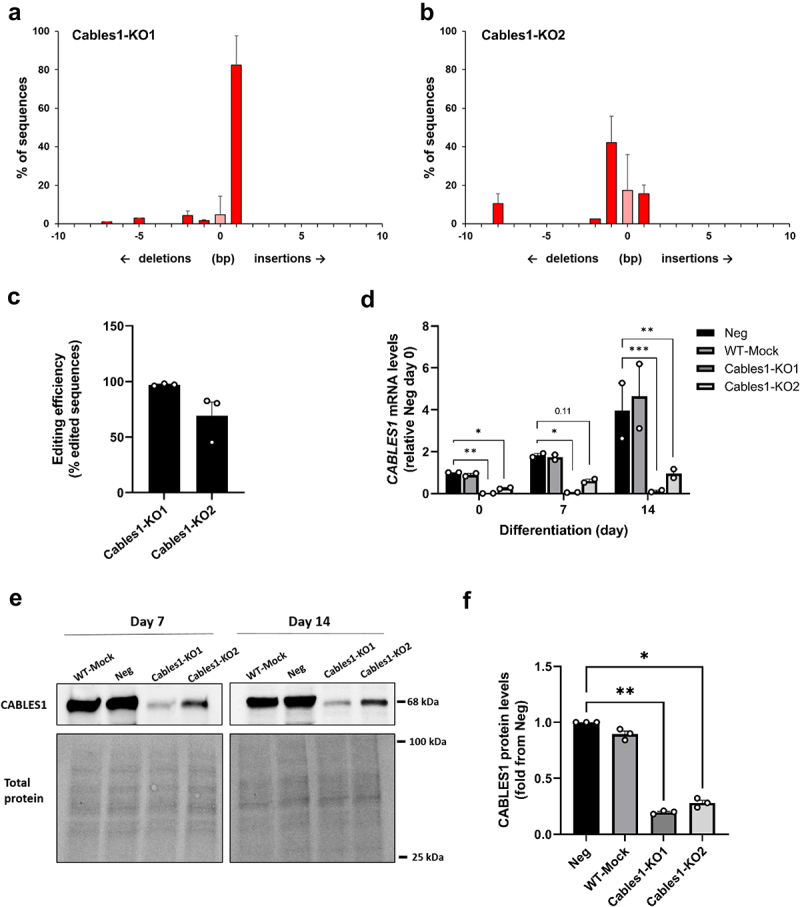
***CABLES1* knockdown in human primary preadipocytes using CRISPR/Cas9 technology**. Indel distributions in the *CABLES1* gene in the knockdown cultures using two different sgRNAs, a) Cables1-KO1 and b) Cables1-KO2, and c) corresponding total gene editing efficiencies as measured by TIDE analysis of Sanger sequencing data. d) Gene expression of *CABLES1* on days 0, 7 and 14 of differentiation in negative control (Neg), mock transfected wild type (WT-Mock) and the two knockdown cultures (Cables1-KO1 and -KO2). *CABLES1* mRNA levels are normalized to *GUSB* as a reference gene, and shown relative to Neg levels on day 0. e) Representative immunoblots of CABLES1 protein levels and total protein (stain-free technology) in WT-Mock, Neg, Cables1-KO1 and Cables1-KO2 cultures after 7 and 14 days of differentiation, and f) quantification on day 14 (*n* = 3). CABLES1 protein levels were normalized to total protein. Data shown as means ± SEM. *p < 0.05, **p < 0.01, ***p < 0.001. *N* = 2–3 independent experiments (different subjects), using triplicates for each experiment.

**Figure 5.**
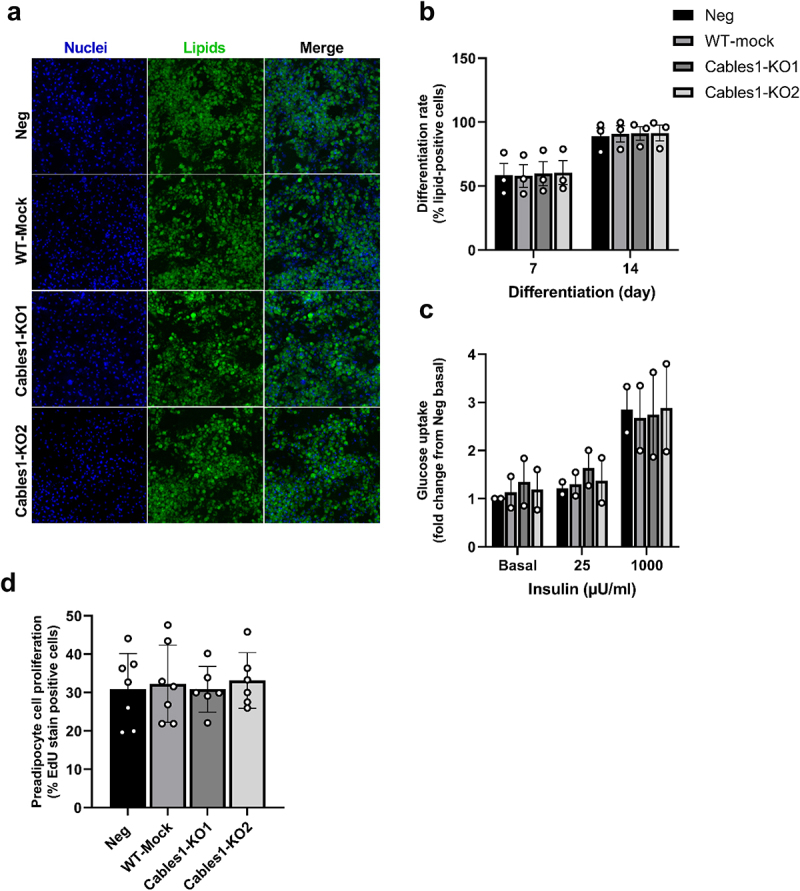
**CABLES1-knockdown did not affect adipocyte differentiation, glucose uptake or proliferation in SVF-derived preadipocytes**. a) Representative images of Neg, WT-Mock, Cables1-KO1 and -KO2 adipocyte cultures on day 14 of differentiation stained with Hoechst nuclear stain (blue) and BODIPY lipid stain (green), and both combined (merge). b) Differentiation rate measured as % lipid positive cells (cells with BODIPY-signal) on day 7 and 14 of differentiation. c) Basal and insulin-stimulated glucose uptake in Neg, WT-Mock and Cables1-KO1 and -KO2 cultures on day 14 of differentiation. d) Proliferation rate of Neg, WT-Mock, Cables1-KO1 and -KO2 preadipocyte cultures measured as percentage of dividing cells (cells with EdU- signal). Data shown as means ± SEM. *N* = 2–3 independent experiments (different subjects), using triplicates for each experiment (except d: 2–6 replicates).

*CABLES1* was successfully knocked down in human primary preadipocytes using our optimized CRISPR/Cas9 protocol. Using two different sgRNAs, named Cables1-KO1 and Cables1-KO2, we achieved a knockdown efficiency of over 95% and 75%, respectively, as measured by Sanger sequencing and TIDE analysis (Figure 4(a-c)). Similar levels of knockdown efficiencies, compared to negative control culture, were also measured on the *CABLES1* mRNA levels throughout differentiation, while WT-mock cultures did not differ from negative control cultures (Figure 4(d)). At the protein level, CABLES1 was lower by 80% and 72% in Cables1-KO1 and -KO2 cultures, respectively, compared to Neg on day 14 of differentiation (Figure 4(e,f)). Ablation of CABLES1 did not affect differentiation rate (Figure 5(a,b)) or basal or insulin-stimulated glucose uptake in differentiated adipocytes (Figure 5(c) and Supplementary Figure S3), nor did it alter cell proliferation rates (Figure 5(d)) or expression of key genes important for adipogenesis or adipocyte function at either day 0, 7 or 14 of differentiation (Supplementary Fig S4).

## Discussion

The aim of this study was to investigate the role of *CABLES1* in SAT in T2D and obesity, and its functional role in adipocyte development and metabolism. Our results demonstrate that *CABLES1* gene expression is downregulated in subjects with T2D and obesity and positively associated with adipocyte glucose uptake, but its ablation in human preadipocytes did not affect adipocyte differentiation or glucose uptake. To our knowledge, this is the first study to characterize *CABLES1*, a gene previously associated with obesity-related traits in GWAS [15,17], in the context of metabolic dysfunction in human AT. Besides being implicated in obesity, *CABLES1* could potentially also play a role in the development of diabetes through its role as an adaptor protein for CDK5, which has been shown to control diabetogenic actions of *PPARG* [13,14], a master regulator of adipogenesis and crucial for adipocyte function [18].

The exploratory data in our study supports an association of *CABLES1* with obesity and T2D. We showed that SAT gene expression of *CABLES1* is downregulated with obesity and T2D, and is negatively associated with markers of hyperglycaemia and obesity, while positively associated with markers of insulin sensitivity, HDL, and adipocyte *ex vivo* lipolysis and glucose uptake, suggesting that higher levels of CABLES1 in SAT are reflecting a metabolically beneficial phenotype. Moreover, in the regression model, the strongest predictor for *CABLES1* gene expression levels was BMI, while WHR and the insulin resistance marker HOMA-IR did not contribute significantly. Besides the associations of *CABLES1* gene expression with clinical parameters, we also observed strong associations with numerous genes important for adipocyte development and function, such as positive correlations with e.g. *PPARG*, and *GLUT4*, which is consistent with subjects having higher levels of *CABLES1* gene expression being more insulin sensitive [19]. Combined, these findings indicate that *CABLES1* might have a beneficial role in promoting a healthy body weight and play an important role in adipocyte development and metabolism.

In order to understand the role of CABLES1 in SAT, which contains many cell types besides adipocytes such as adipocyte precursor cells and immune cells [20,21], we investigated which cell types in SAT express *CABLES1* and, hence, would be the driver of the observed correlations. Our findings show that the CABLES1 protein is essentially found only in adipocytes and not in the SVF, which contains most other SAT cell types. At the gene level, expression was also found in preadipocytes and some immune cells such as macrophages, although expression was clearly enriched in adipocytes. However, the proportion of other SAT resident cells, such as immune cells, can vary greatly depending on e.g. obesity status and related SAT inflammation [21]. Thus it is possible that conditions which alter the proportions of SAT-resident cells could affect *CABLES1* levels.

To further investigate the role of CABLES1 in SAT, and adipocytes being the main expressing cell type, we performed functional assessments of CABLES1 in human adipocyte development and metabolism, using CRISPR/Cas9 knockdown in human primary preadipocytes. We have previously demonstrated that deleting genes in human preadipocytes with CRISPR/Cas9 is a good model for studying cell cycle and adipocyte function factors [10,22]. We found that ablation of *CABLES1* in human preadipocytes caused no phenotypical changes during adipogenesis or in the mature adipocytes, in regards to gene expression of key genes e.g. *ADIPOQ*, *CD36*, *PPARG*, differentiation rate or glucose uptake. None of the analysed genes were affected by ablation of *CABLES1* in the *in vitro* setting. This was somewhat surprising given that *CABLES1* in SAT correlated strongly with many of these genes. Moreover, CABLES1 has been shown to activate CDK5 [5], which in turn can lead to PPARG phosphorylation and subsequent changes gene expression levels, such as the *in vivo* insulin-sensitizing adipokine *ADIPOQ*, in adipocytes, but without affecting adipogenic capacity [13]. We did see a positive correlation between *CDK5*, but not *ADIPOQ*, and *CABLES1* gene expression in SAT in our study, but given the lack of phenotypic effect of CABLES1 ablation in adipocytes, it was beyond the scope of this study to explore this further.

We also observed that *CABLES1* mRNA in adipocytes is downregulated following induction with the adipogenic cocktail. This may be caused by the presence of IBMX in the medium during the first 5 days of culture, but similar effects has previously also been shown for other cyclins and CDKs [12], suggesting that its downregulation could be involved in the early steps of the adipogenic process, of which cell cycle arrest is an important step [23], and CABLES1 has been shown to act as a negative regulator of the cell cycle [24]. Although we observed correlations between the expression of *CABLES1* and a number of cell cycle-regulating genes, ablation of *CABLES1* in human preadipocytes did not correlate with e.g. *CDK2* or affect cell proliferation or differentiation in our study, suggesting that other cell cycle regulating factors are more important than *CABLES1* in this cell type.

Overall, the results from our functional assessments of *CABLES1* in adipocytes suggest that the metabolic impairments, as seen in SAT in obesity and T2D, may not be caused by decreased adipocyte *CABLES1* levels directly, but rather that decreased levels of this gene could be a consequence of metabolic impairment caused by other factors, but more studies are needed to entangle this. It should also be considered that *the in vitro* knockdown of *CABLES1*, does not necessarily reflect the *in vivo* complex metabolic regulation in human adipose tissue.

This study has some limitations. The number of subjects in this study was limited, and although we included a validation cohort, increasing the number of subjects would have provided more robust conclusions, as not all correlations could be validated. The functional studies included only preadipocytes from women, and a limited number of experiments. Even though our multivariate regression model did not suggest a sex difference of *CABLES1* gene expression in SAT, future studies should ideally include preadipocytes also from men. Moreover, the functional assessments were performed in *in vitro* differentiated adipocytes using a limited number of assays to detect alterations in adipocyte function, whereas the correlations were with adipose tissue that contains many cell types besides adipocytes, such as immune cells. Even though our findings indicate that adipocytes are the major CABLES1 expressing cell type in adipose tissue, other cells might to some degree drive the correlations. Considering the comparatively low levels of expression in these other cell types, this contribution is likely small.

In conclusion, our findings suggest that *CABLES1* gene expression downregulation in SAT in subjects with T2D and obesity may be secondary to metabolic dysregulation, possibly as a protective mechanism. Although we cannot completely rule out the possibility that *CABLES1* plays a causal role in adipose tissue function or dysfunction *per se*, our data indicate that it is dispensable for adipogenesis, and that loss of this gene does not cause any impairment in glucose uptake or key genes involved in glucose uptake and lipid storage, but further studies are warranted. Our study also highlights the importance of functional evaluation of candidate genes, implicated in studies identifying disease and phenotype-associated genes, in order to establish causality.

## Methods

### Subjects, adipose tissue biopsies and handling

*Cohort 1* included 19 subjects with T2D and 20 without T2D. The participants from both groups were matched for age and body mass index (BMI) (20 women/19 men; age: 34–72 years; BMI: 22.4–39.9 kg/m^2^). Subjects underwent an oral glucose tolerance test (OGTT), body composition was measured using whole-body magnetic resonance imaging (MRI), and SAT biopsies were obtained by needle aspiration of the abdomen after local dermal anaesthesia with lidocaine (Xylocain; AstraZeneca, Sweden). Subjects´ clinical characteristics, study design and methods have previously been described in detail [25]. SAT biopsies from *cohort 1* were used for RNAseq analysis to investigate correlations of *CABLES1* gene expression with clinical characteristics and expression of genes relevant for adipose tissue, and for group comparison of *CABLES1* gene expression between subjects with and without T2D, and between women and men.

*Cohort 2* included 102 subjects without T2D and with a wide distribution of BMI and insulin sensitivity (65 women/26 men; age: 18–72 years; BMI: 20.4–58.2 kg/m^2^; HOMA-IR: 0.58–14.1). SAT samples were obtained by needle biopsy from 80 subjects as described for *cohort 1*. In addition, paired samples of SAT and omental adipose tissue (OAT) were obtained during surgery from a subgroup of healthy subjects undergoing kidney donation (*n* = 7) at the Sahlgrenska University Hospital, as well as bariatric surgery (*n* = 15) at the Uppsala University Hospital. SAT biopsies from *cohort 2* were used to validate association findings from *cohort 1*, as well as for group comparisons between women and men. SAT and OAT samples were used for group comparisons of *CABLES1* expression between lean, overweight and obese subjects. SAT from eight subjects were used for establishing primary preadipocyte cultures for temporal profiling of *CABLES1* gene expression during differentiation into adipocytes.

*Cohort 3* included 12 subjects (9 women/3 men, A: 31–61 years, BMI: 31.1-40.1 kg/m^2^) with obesity and T2D who underwent Roux-en-Y gastric bypass (RYGB) surgery at the Department of Surgery at Uppsala University Hospital. Inclusion criteria were 18–60 years old, a BMI of 30–45 kg/m^2^, T2D for a maximum of 10 years, and treated with a maximal of three oral antidiabetic agents, excluding insulin. Data and SAT needle biopsies were obtained at baseline (pre-surgery) and 4, 24, and 104 weeks after RYGB surgery. A detailed description of the study design, methods and clinical characteristics of the subjects have been reported previously [26,27]. Clinical characteristics for baseline values are show in Table 1. SAT biopsies from *cohort 3* were used to investigate the effects of weight loss on *CABLES1* gene expression and for group comparisons between lean, overweight and obese subjects (qPCR).

For all cohorts, subjects with type 1 diabetes, other endocrine disorders, cancer or other major illnesses, as well as ongoing medication with beta-adrenergic blockers, systemic glucocorticoids or immune-modulating therapies, were excluded from the study. Fasting blood samples were collected for blood chemistry analysis at the Department of Clinical Chemistry at the respective hospitals. Clinical characteristics of the cohorts are shown in Table 1.

One part of adipose tissue samples was snap-frozen in liquid nitrogen and used for gene and protein expression analysis, and one part was used for adipocyte and stromal vascular fraction (SVF) isolation. Isolated mature adipocytes were used for adipocyte size measurements, and *ex vivo* analyses of lipolysis and glucose uptake, as previously reported [25,28]. Not all analysis were performed for all subjects due to limited amount of adipose tissue.

3 additional SAT biopsies (not included in any of the cohorts) obtained from healthy subjects without T2D (3 women; age: 26–45 years; BMI 21.6–37.3 kg/m^2^), were used for CRISPR/Cas9 *CABLES1* knockdown experiments in preadipocytes

The study protocols were approved by the Regional Ethics Review Boards in Gothenburg (Dnr 336–07) and Uppsala (Dnr 2013/330 and Dnr 2013–183/494). All methods were performed in accordance with relevant guidelines and regulations. Written informed consent was obtained from all study participants.

### Gene expression

#### RNAseq

Adipose tissue samples from *cohort 1* were used for targeted analysis of selected genes involved in adipocyte glucose and lipid metabolism (see Supplementary File 2 for gene expression data) obtained with RNAseq (Exiqon A/S, Vedbaek, Denmark) as reported before [25]. Total RNA was isolated from 5 mg starting material to obtain 100 ng RNA for sequencing and 60 ng for quality control. RNA quality was assessed by Nanodrop absorbance measurements (OD260/230 and OD26/280) and RNA integrity measurements by an Agilent Bioanalyzer. PolyA RNA selection & library preparation was done using an Oligo-dT magnetic bead system according to Illumina protocol. Library QC was done using an Agilent Bioanalyzer. Sequencing was done using Illumina instrument with a 100bp paired-end Read length. QC of raw data encompassed adaptors trimming, Q-score distribution, and distribution of reads per sample. mRNA expression data are shown as Fragments Per Kilobase of transcript per Million (FPKM).

#### Quantitative real-time PCR

Adipose tissue from *cohorts 2–3*, and preadipocytes and adipocytes from *in vitro* cultures were used for qPCR analysis. Total RNA was extracted from adipose tissue or cells using the RNeasy lipid tissue mini kit (Qiagen, Hilden, Germany) according to the manufacturer’s instructions. RNA concentrations were measured using Nanodrop (Thermo Scientific) and RNA was then reverse transcribed using High Capacity cDNA reverse transcriptase kit (Applied Biosystems, Foster City, CA, USA). Gene expression analysis was performed using qPCR and TaqMan gene expression assays (listed in Supplementary Table S1) on the QuantStudio3 sequence detection system (Applied Biosystems). Data were analysed using a 2^−delta Ct^ method and presented as relative quantification using glucuronidase beta (*GUSB)* as a reference gene. Samples were run in duplicates.

### Isolation of adipocytes and preadipocytes from SAT

Adipocyte and SVF isolation from SAT biopsies were performed as previously described [22]. In brief, adipose tissue was digested with collagenase, and the SVF, containing preadipocytes, was separated from mature adipocytes into a Falcon tube. The mature adipocytes were used for fat cell size, *ex vivo* glucose uptake and lipolysis assessments, as previously reported [25]. The SVF was centrifuged at 1200 RPM for 3 minutes, and the pellet was cultured in preadipocyte medium: DMEM/F12 medium containing 10% foetal calf serum (FCS) (Invitrogen), 100 units/ml penicillin and 100 g/ml streptomycin (PEST, Life Technology), 0.04 mg/ml gentamycin (Gibco) and 17 ng/ml basic fibroblast growth factor (bFGF) (Sigma) at 37°C. Media was replaced after every two days. After the cells reached about 70% confluence, they were trypsinized and frozen in DMEM-F12 with 20% FCS, 10% dimethyl sulphoxide (DMSO) at − 150°C until further culturing and analysis.

### Preadipocyte culturing and differentiation

Preadipocytes were cultured and differentiated as reported before [22]. Preadipocytes from passage 1 were thawed at 37°C and expanded into a *T*-75 flask using preadipocytes medium. Upon reaching 70% confluence, the cells were trypsinized and seeded again into a 12 well plate at density 15,000 cells/cm (passage 2) using preadipocytes media. After reaching confluence, adipogenesis was induced by adding a differentiation cocktail DMEM-F12, 1% PEST, 100 nM insulin, 17 µM pantothenate (Sigma), 33 µM biotin (Sigma), 1 µM dexamethasone (Sigma), 1 µM rosiglitazone (Sigma), 250 µM 3-isobutyl-1-methylxanthine (IBMX, Sigma), 10 µg/ml transferrin (Sigma), 2 nM triiodothyronine (T3, Sigma) for 5 days, changing the medium on day 3. The differentiation continued using maintenance media (composition as that of differentiation cocktail except for omitting IBMX) for 14 days to obtain mature adipocytes. Medium was replenished every 2–3 days. *CABLES1* gene expression was measured in cells collected upon confluence (day 0) and days 2, 4, 8 and 14 post induction for temporal profiling, or, for gene editing experiments (see below), on days 0, 7 and 14 for gene edited cells.

### Western-blot

Total protein was extracted from adipocytes and stromal vascular fraction (SVF) isolated from SAT, and *in vitro* differentiated adipocytes on day 7 and 14 of differentiation. Cells were lysed in ice-cold lysis buffer: 25 mM Tris-HCl (Sigma), pH 7.4; 0.5 mM EGTA (Sigma); 25 mM NaCl (Sigma); 1% Nonidet *P*-40; 10 mM NaF; 100 nM okadaic acid (Alexis Biochemicals), 1X Complete protease inhibitor cocktail (Roche, Indianapolis, IN, USA) and 1 mM orthovanadate (Sigma). Lysates were vortexed and incubated on ice for 10 minutes and then centrifuged at 15,000 g for 15 minutes at 4°C. The infranatant was transferred into a new tube and saved at −80°C. Protein concentration was determined using a BCA protein assay kit (Pierce, Thermo Scientific). 10 µg proteins were separated by SDS-PAGE (5–8% gradient stain-free gels; BioRad), transferred to nitrocellulose membranes and blocked with 0.05% tween-PBS with 5% BSA. Membranes were incubated overnight with the primary antibody anti-CABLES1 (1:240, HPA073649; Sigma-Aldrich). Membranes were washed with 0.05% tween-PBS and incubated for 1 hour at room temperature with horseradish peroxide-conjugated secondary antibody (anti-rabbit, 1:2000; Cell Signalling). Protein bands and stain-free blot images were detected using enhanced chemiluminescence and the ChemiDocTM MP System (Biorad) and quantified using the Image Lab Software (Version 6.1.0; BioRad).

### CRISPR/Cas9 gene editing of human preadipocytes

The *CABLES1* gene was knocked down in human SVF-derived preadipocytes from three healthy subjects using CRISPR/Cas9 technology as previously described [22], and gene expression, proliferation rate, differentiation rate and glucose uptake assessments [10,29] were made during and after differentiation into mature adipocytes for 14 days (see Supplementary File 1 for a detailed description of the methods).

### Statistics

All data were analysed for normality using the Shapiro-Wilk test and by analysing histograms. Log-transformations of data where performed when appropriate. Spearman's correlation tests were performed between *CABLES1* mRNA expression levels in SAT and markers of insulin resistance and obesity, and expression of genes (targeted analysis). Significant group differences were assessed using an unpaired Student’s t-test, and comparisons between multiple groups were performed using one-way ANOVA or mixed effect analysis. Multiple comparisons were corrected for the false discovery rate using the original Benjamini-Hochberg method. Statistical analyses were performed with IBM SPSS Statistics 28 and GraphPad Prism 9 software. A p-value <0.05 was considered statistically significant. Data are shown as means ± SEM unless indicated otherwise. ‘N’ refers to the number of individual subjects. Number of replicates for each analysis of the CRISPR/Cas9 experiments are indicated in the relevant Method sections and in Figure legends.

## Supplementary Material

Supplemental Material

## Data Availability

Some or all datasets generated during and/or analysed during the current study are not publicly available but are available from the corresponding author on reasonable request.
