# Supplementary material for: CABLES1 expression is reduced in human subcutaneous adipose tissue in obesity and type 2 diabetes but may not directly impact adipocyte glucose and lipid metabolism
Source: Adipocyte. 2023 Aug 9;12(1):2242997. doi: 10.1080/21623945.2023.2242997 (PMC10413912; doi:10.1080/21623945.2023.2242997)
Supplement: Supplemental Material [file KADI_A_2242997_SM1924.zip › CABLES1_Supplementary_File1_final__1_.docx]

**SUPPLEMENTARY FILE 1**

***CABLES1* expression is reduced in human subcutaneous adipose tissue in obesity and type 2 diabetes but may not directly impact adipocyte glucose and lipid metabolism**

Susanne Hetty^1*^, Milica Vranic^1^, Prasad G Kamble^2^, Martin H Lundqvist^1^, Maria J Pereira^1^, Jan W Eriksson^1^

^1^Department of Medical Sciences, Clinical Diabetes and Metabolism, Uppsala University, Uppsala, Sweden

^2^ Innovation Strategies & External Liaison, Pharmaceutical Technologies & Development, AstraZeneca R&D, Mölndal, Sweden

**Supplementary methods**

***Enrichment prediction scoring***

We leveraged enrichment scores previously published across the cell types within adipose tissue^1^. In brief, the enrichment score is the mean correlation between gene expression and the expression of three reference transcripts selected to represent specifically each cell type. We extracted *CABLES1* enrichment data for all cell types found in visceral (VAT) and subcutaneous adipose tissue (SAT) in the R statistical software version 4.3.0^2^. To visualize the enrichment prediction scores across cell types and to see in which *CABLES1* expression is enriched we drew lollipop lots with ggplot2 v. 3.4.2^3^ and gridExtra v.4.3.0^4^ R packages. Non-positive score values were set to 0, a dot defined a positive correlation score and a simultaneous positive correlation with all three transcripts defined gene expression as cell-type enriched and was depicted with a larger circle symbol. A mean correlation value with the adipocyte cell type is defined by correlation with the expression of the three adipocyte reference transcripts: ADIPOQ, LIPE, and PLIN1.

***CRISPR/Cas9 gene editing of human preadipocytes***

The *CABLES1* gene was knocked out in in human SVF-derived preadipocytes from three different subjects using CRIPSR/Cas9 technology. Two single guide RNAs (sgRNA) Cables1-KO1 and Cable1-KO2 (5´-ACATGCGGCAACACGATACCagg-3´ and 5´-TATCGCGACAGTACCCAAGTcgg-3´ respectively) (PAM sequence in lowercase letters), targeting exon three and four of the *CABLES1* gene, which are common for all isoforms and containing important functional domains, were designed using the online tool https://chopchop.cbu.uib.no/ (2021–03-01). CRISPR/Cas9 components were delivered into the cells using modified synthetic sgRNAs and and TrueCut Cas9 protein v2 as a ribonucleoprotein complex and electroporation with the Neon Transfection system (all from Thermo Fisher, MA, USA) using our optimized human preadipocytes protocol as previously reported ^5^. Control cultures were either electroporated without any Cas9 protein or sgRNA (WT-Mock) or using a sgRNA targeting the safe harbour locus AAVS1 (Thermo Fisher) as a negative control (Neg), as previously reported ^6,7^. After transfection, preadipocytes were cultured in preadipocytes medium without antibiotics. Antibiotics were re-introduced into the preadipocytes medium 48 hours post transfection. Knockdown efficiency was estimated by gene expression (day 0, 7 and 14 of differentiation), protein levels on day 7 and 14, and DNA Sanger sequencing of PCR-amplified DNA segments spanning the CRISPR-Cas9 cut site (PCR primer sequences: Cables1-KO1 forward primer, 5’-AGCAGGCAACTGACTGTGTT-3’ and reverse primer 5’- GTTCAGGCGCATATCTGGGA-3’; Cables1-KO2 forward primer 5’- GTGGGAGCGTGCAAATACTG-3’ and reverse primer 5’- CCTGACTTCGATACCGGTGG-3’) followed by chromatogram analysis using the online tool Synthego Performance Analysis, ICE Analysis. 2019. v3.0. Synthego; [2021-04-29].

***Phenotypic assays for* in vitro *differentiated adipocytes***

**Proliferation assay**

Cell proliferation was measured using the Click-iT EdU Cell Proliferation Kit for Imaging, Alexa Fluor 555 dye (Invitrogen) according to the manufacturer’s instructions. Cells were plated on a 12-well plate format at 15000 cells/cm^2^ and proliferation rates were assessed on days 1-6 after plating (not all cultures were asses at all time points). At each time point half of the medium was replaced with 2x EdU solution (final concentration 10 μM) and the cells were incubated for 6 hours at 37 °C. After incubation, the medium was removed and cells were fixed in 4 % formaldehyde for 15 minutes and washed twice with 3 % BSA in PBS. Cells were then incubated with 0.5 % Triton®X-100 in PBS for 20 minutes for membrane permeabilization, and washed twice with 3 % BSA in PBS. Click-iT® Plus reaction cocktail was added for the incubation period of 30 minutes at room temperature, protected from light. Cells were washed with PBS and incubated with nuclear stain (Hoechst® 33342, 5 μg/mL) for 30 minutes at room temperature. After washing twice with PBS, cells were imaged using ImageXpress Pico automated cell imaging system. Image analyses of proliferation rates were done using CellReporterXpress software, measured as the percentage of EdU positive cells. Data is shown as mean percentage positive cells i.e. number of dividing cells. Each condition (preadipocyte cultures of Neg, Mock-WT, Cables1-KO1 and -KO2) were measured for 2-6 time points for each independent experiment (n=3).

**Differentiation rate**

Differentiation rate and lipid content were measured on day 7 and 14 of differentiation as previously described ^4^. In short, cells were washed with PBS and fixed with 4% formaldehyde (Histolab, Gothenburg) and stained with the fluorescent dyes BODIPY 493/503 (Molecular Probes, OR), for neutral lipid staining, and the nucleic stain Hoechst 33342 (Invitrogen, MA, USA). Cells were imaged on the ImageXpress Pico automated cell imaging system (Molecular Devices). Image acquisition settings were 10x magnification, and a 6 × 6 square image scan covering approximately 20% of the total area of the center of each well. Differentiation rate was calculated as the percentage of lipid-positive cells using the automated cell scoring software function (CellReporterXpress software), which identifies cells that either contains lipids (cells with positive signal for Bodipy and Hoeschst stain) or that do not contain any lipids (cells with Hoeschst stain signal but no Bodipy signal). Fluorescence background subtraction is performed by the software and uniformly applied to all images. All images were also manually inspected for irregularities. Each condition (Neg, Mock-WT, Cables1-KO1 and -KO2) were run in triplicates for each independent experiment (n=3).

**Glucose uptake**

Glucose uptake assay were performed on adipocytes differentiated for 14 days (n = 2) using the luminescence Glucose Uptake-GLO kit (Promega) according to the manufacturer’s instructions and as described previously ^3,4^. In brief, cells were washed 2 times with warmed PBS and then incubated for 2 h in Krebs Ringer HEPES (KRH) buffer containing 0.01% BSA (Sigma), with 5 mM glucose (Sigma), 200 nM adenosine (Sigma), and pH 7.4. Cells were then washed with KRH without glucose 2 times, followed by incubation for 30 minutes at 37 °C in KRH without glucose without or with two different concentration of insulin (25 μU/ml or 1000 μU/ ml) to assess basal and physiological and maximal insulin-stimulated glucose uptake. The glucose analog 2-deoxy-2-glucose (2DG, final concentration 1 mM) was added for the last 10 min of the incubation. The reaction was terminated by adding Stop buffer, followed by a Neutralization buffer provided in the kit. Detection was performed using the manufacturer's instructions. Sample and Detection reagent were mixed in a 1:1 ratio and incubated for 1-2 hours before luminescence reading on a plate reader (SpectraMax® iD3, Molecular Devices). Glucose uptake was calculated as the relative amount of accumulated 2DG6P in the cells, normalized to total protein amount in each well. Each condition (Neg, Mock-WT, Cables1-KO1 and -KO2) were run in triplicates for each independent experiment (n=2).

**Supplementary table 1.** Taqman probes used for qPCR quantification of gene expression

| **Gene name** | **Abbreviation** | **TaqMan probe** |
| --- | --- | --- |
| Adiponectin | *ADIPOQ* | Hs00605917 |
| Adipose triglyceride lipase | *ATGL, PNPL1* | Hs00982042 |
| α-2A adrenergic receptor | *ADRA2A* | Hs01099503 |
| CCAAT eenhancer binding protein alpha | *CEBPA* | Hs00269972 |
| Cdk5 and Abl enzyme substrate 1 | *CABLES1* | Hs01106667 |
| Cluster of differentiation 36 | *CD36* | Hs00354519 |
| Fatty acid synthase | *FASN* | Hs01005622 |
| Glucose transporter 1 | *GLUT1, SLC2A1* | Hs00892681 |
| Glucose transporter 4 | *GLUT4, SLC2A4* | Hs00168966 |
| Glucuronidase beta | *GUSB* | Hs00939627 |
| Hormone-sensitive lipase | *HSL, LIPE* | Hs00193510 |
| Lipoprotein lipase | *LPL* | Hs00173425 |
| Peroxisome proliferator-activated receptor gamma | *PPARG* | Hs01115513 |

**Supplementary Figure S1.**


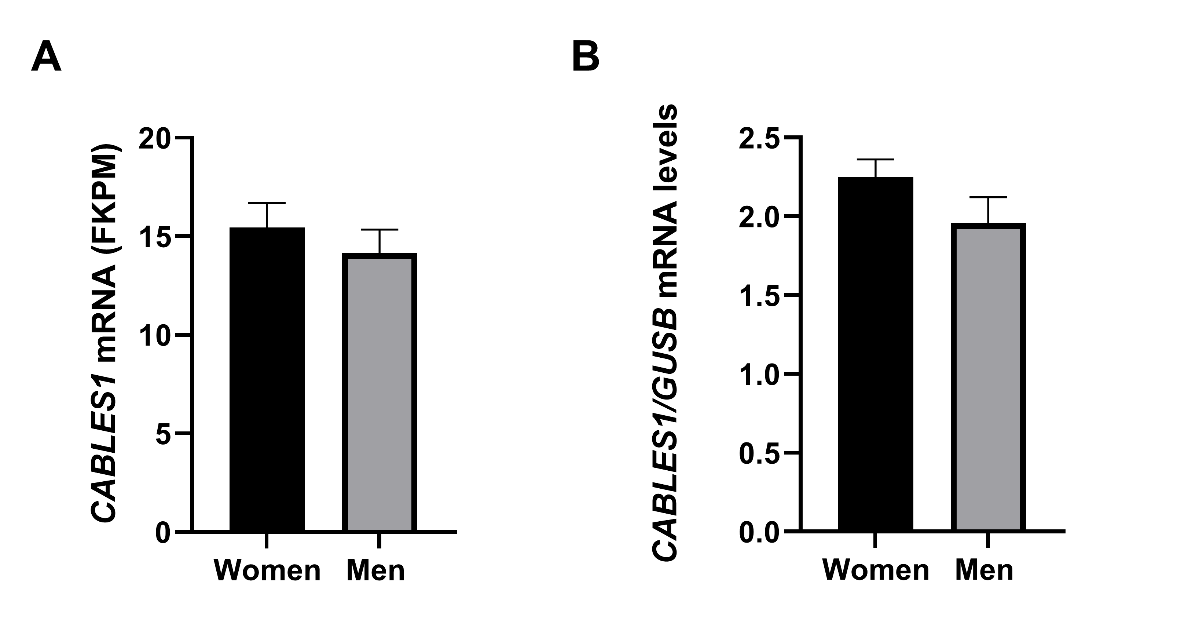


**Supplementary Figure S1 legend. *CABLES1* gene expression in SAT in men and women.** Gene expression levels of *CABLES1* in **A)** *cohort 1* (n=20/19; women/men), and **B)** *cohort 2* (n=26/65, men/women) measured by RNAseq and qPCR, respectively. pPCR data were normalized using *GUSB* as a reference gene. All data are shown as mean ± SEM.

**Supplementary Figure S2.**


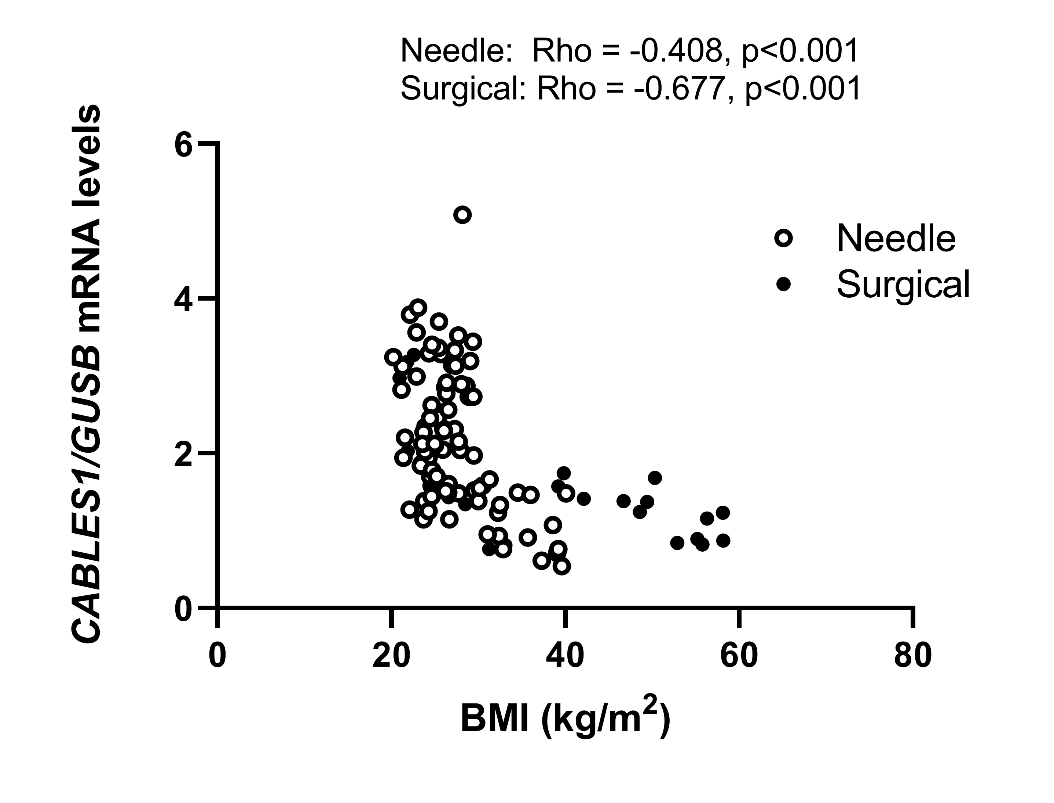


**Supplementary Figure S2 legend.** CABLES1 gene expression levels in SAT obtained from needle and surgical biopsies correlated with BMI (*cohort 2*). Gene expression measured by qPCR and data normalized to GUSB as a reference gene. N=91. Figure shows Spearman’s rho correlation coefficients.

**Supplementary Figure S3.**


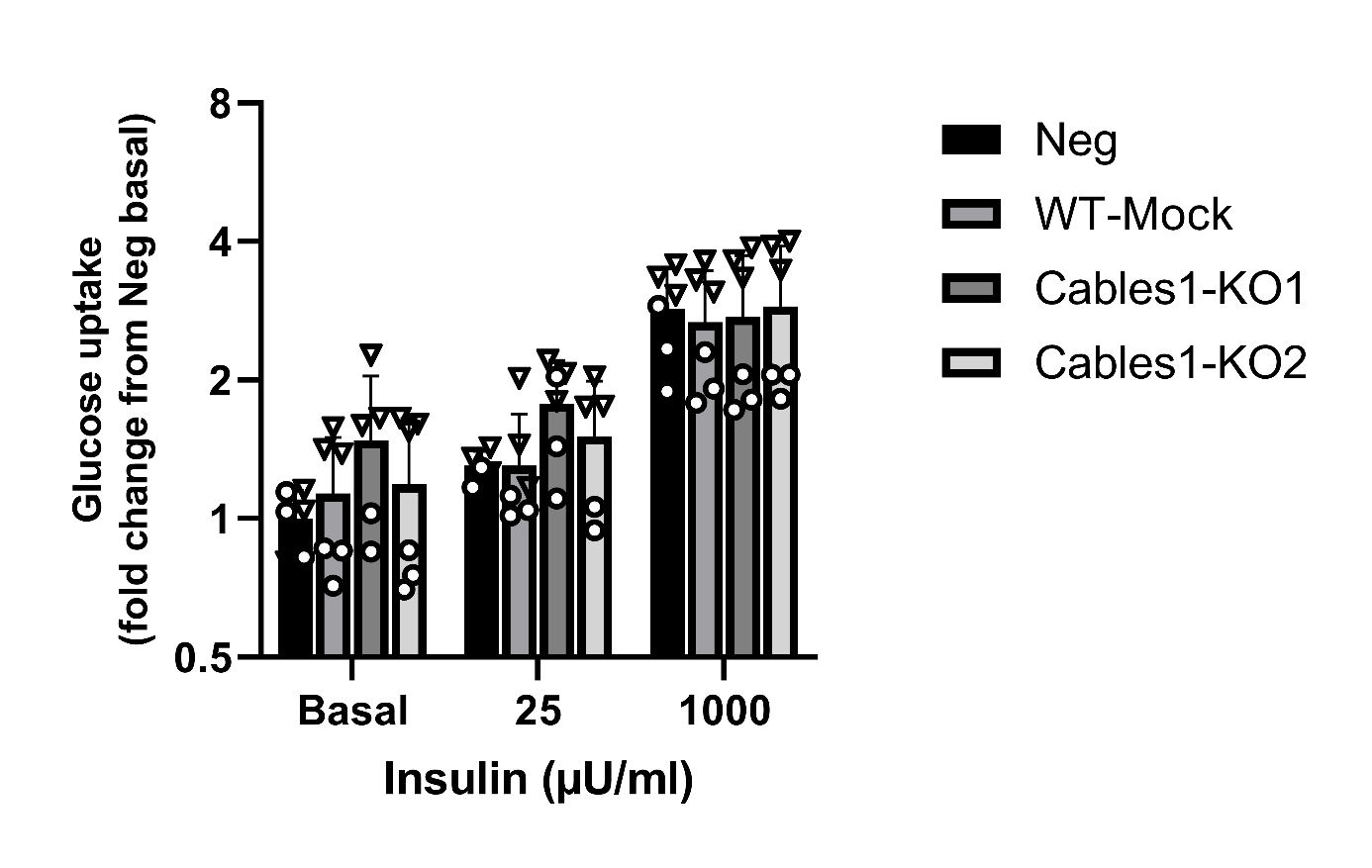


**Supplementary Figure S3 legend** Basal and insulin-stimulated glucose uptake in Neg, WT-Mock and Cables1-KO1 and -KO2 cultures on day 14 of differentiation. Data shown as means ± SEM. N= 2 independent experiments (different subjects), using triplicate cultures for each experiment (triplicate values from each subject indicated by open triangles or circles)

**Supplementary Figure S4.**


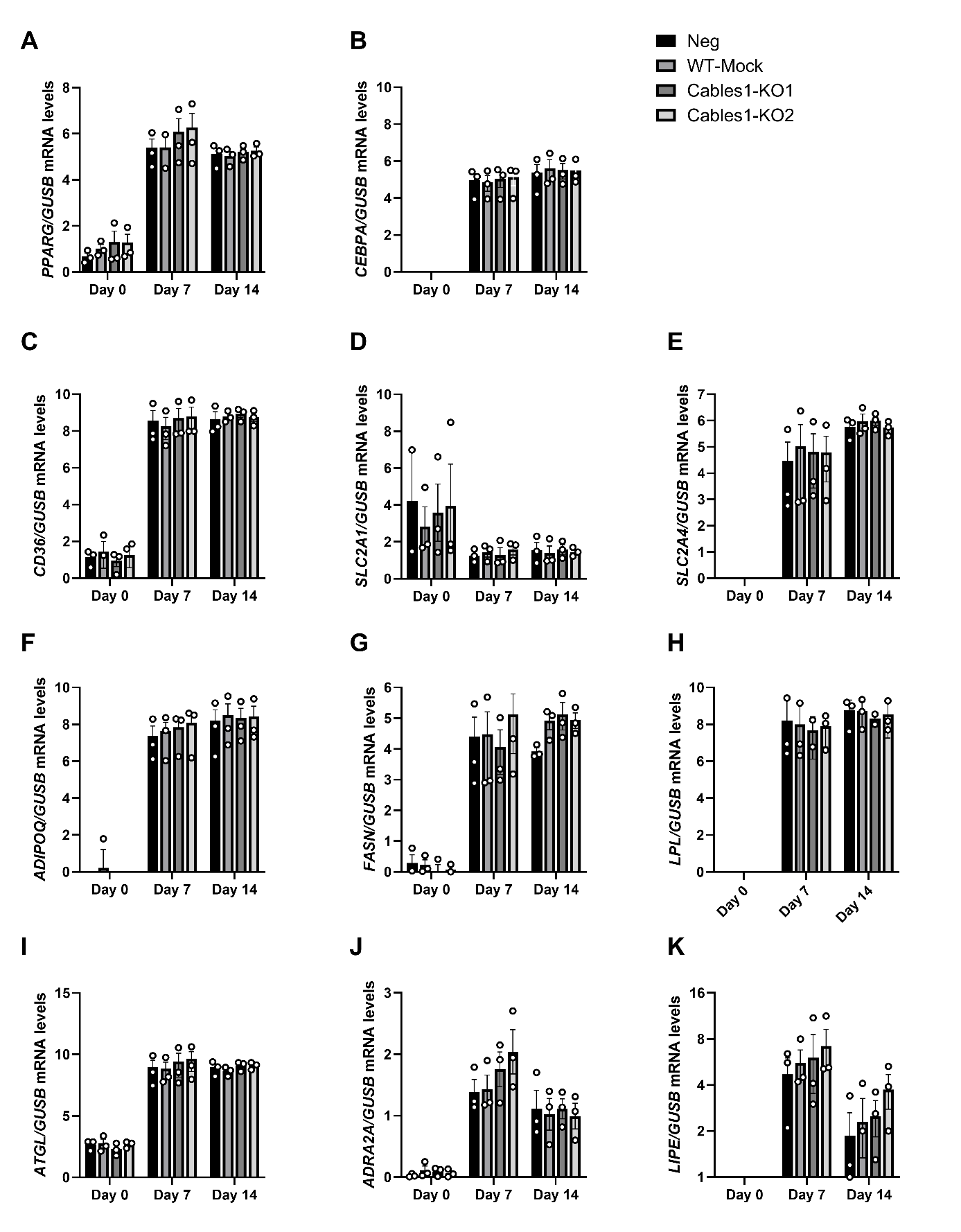


**Supplementary Figure S4 legend. Gene expression levels of key genes for adipocyte function are not altered by *CABLES1* deficiency during adipogenesis.** Gene expression of A) *PPARG*, B) *CEBPA*, C) *CD36*, D) *SLCA1*, E) *SLCA4*, E) *ADIPOQ*, F) *FASN*, G) *LPL*, I) *ATGL*, J) *ADRA2A* and K) *LIPE* in Neg, Mock, Cables1-KO1 and Cables1-KO2 in human preadipocyte cultures on day 0,7 and 14 of differentiation. mRNA levels normalized to *GUSB* as a reference gene. Data log transformed and shown as means ± SEM. N=3 independent experiments (different subjects), and three replicates for each subject.

**References**

1. Norreen-Thorsen, M. *et al.* A human adipose tissue cell-type transcriptome atlas. *Cell reports* **40**, 111046, doi:10.1016/j.celrep.2022.111046 (2022).
2. R Development Core Team (2011). R: a language and environment for statistical computing [Internet]. http://www.R-project.org/. 2015. Available from: <http://www.R-project.org/>.
3. ggplot2: Elegant Graphics for Data Analysis (3e) [Internet]. [cited 2023 Jun 9]. Available from: <https://ggplot2-book.org/>
4. Auguie B, Antonov A. gridExtra: Miscellaneous Functions for “Grid” Graphics [Internet]. 2017 [cited 2023 Jun 9]. Available from: <https://cran.r-project.org/web/packages/gridExtra/index.html>
5. Kamble, P. G. *et al.* Proof-of-concept for CRISPR/Cas9 gene editing in human preadipocytes: Deletion of FKBP5 and PPARG and effects on adipocyte differentiation and metabolism. *Scientific reports* **10**, 10565, doi:10.1038/s41598-020-67293-y (2020).
6. Ahmed, F. *et al.* ESR2 expression in subcutaneous adipose tissue is related to body fat distribution in women, and knockdown impairs preadipocyte differentiation. *Adipocyte*, doi:10.1080/21623945.2022.2102116 (2022).
7. Pereira, M. J. *et al.* CDKN2C expression in adipose tissue is reduced in type II diabetes and central obesity: impact on adipocyte differentiation and lipid storage? *Transl Res* **242**, 105-121, doi:10.1016/j.trsl.2021.12.003 (2022).
